# Increased Standardised Incidence Ratio of Malignant Pleural Mesothelioma in Taiwanese Asbestos Workers: A 29-Year Retrospective Cohort Study

**DOI:** 10.1155/2015/678598

**Published:** 2015-07-28

**Authors:** Cheng-Kuan Lin, Yu-Ying Chang, Jung-Der Wang, Lukas Jyuhn-Hsiarn Lee

**Affiliations:** ^1^Department of Public Health, College of Medicine, National Cheng Kung University, Tainan 70101, Taiwan; ^2^Division of Environmental Health and Occupational Medicine, National Health Research Institutes, 35 Keyan Road, Zhunan, Miaoli County 35053, Taiwan; ^3^Departments of Internal Medicine, Occupational and Environmental Medicine, National Cheng Kung University Hospital, Tainan 70403, Taiwan; ^4^Department of Environmental and Occupational Medicine, National Taiwan University Hospital, Taipei 10002, Taiwan; ^5^Institute of Occupational Medicine and Industrial Hygiene, College of Public Health, National Taiwan University, Taipei 10020, Taiwan

## Abstract

*Objective*. This paper aimed to determine the standardised incidence ratio (SIR) of malignant pleural mesothelioma (MPM) in workers exposed to asbestos in Taiwan. *Methods*. All workers employed in asbestos-related factories and registered by the Bureau of Labour Insurance between 1 March, 1950, and 31 December, 1989, were included in the study and were followed from 1 January, 1980, through 31 December, 2009. Incident cases of all cancers, including MPM (ICD-9 code: 163), were obtained from the Taiwan Cancer Registry. SIRs were calculated based on comparison with the incidence rate of the general population of Taiwan and adjusted for age, calendar period, sex, and duration of employment. *Results*. The highest SIR of MPM was found for male workers first employed before 1979, with a time since first employment more than 30 years (SIR 4.52, 95% CI: 2.25–8.09). After consideration of duration of employment, the SIR for male MPM was 5.78 (95% CI: 1.19–16.89) for the workers employed for more than 20 years in asbestos-related factories. *Conclusions*. This study corroborates the association between occupational asbestos exposure and MPM. The highest risk of MPM was found among male asbestos workers employed before 1979 and working for more than 20 years in asbestos-related factories.

## 1. Introduction

Asbestos is the collective name for a group of fibrous silicate minerals that are widely used in many industries due to their strength and fire-resistant properties [1]. Asbestos can be divided into serpentine (chrysotile) and amphibole, with exposure to different types associated with different degrees of health risk. Chrysotile accounts for 95% of the asbestos in use around the world and is the only type of asbestos in commercial use today [2]. All forms of asbestos cause malignant mesothelioma and lung, laryngeal, and ovarian cancers and may cause gastrointestinal and other cancers [3].

All forms of asbestos have been classified as carcinogens by the World Health Organization (WHO) [4] and as group I human carcinogens by the International Agency for Research on Cancer (IARC) [5]. The WHO recently estimated that 125 million people globally are exposed to asbestos in the workplace, and about 107,000 people die each year from asbestos-related diseases resulting from occupational exposure [6]. Asbestos-related diseases include benign lesions of asbestosis, pleural effusion, pleural plaques, diffuse pleural thickening, and malignancies of the lung, peritoneum, and pleura [7]. Malignant mesothelioma (MM) is the primary malignant neoplasm arising from the serosal membranes (mesothelium), the protective linings that cover many of the body's internal organs [8], and the pleura is the most frequent site, also termed as malignant pleural mesothelioma (MPM) [9]. The association between MPM and asbestos exposure was observed by Wagner et al. in 1960 [10]. MM is a fatal disease with a median survival of approximately nine months, and it occurs with a long latency of 20–40 years after exposure to asbestos. It has been estimated that the population attributable fraction of MM among men from occupational exposure to asbestos is approximately more than 80% [11].

A few studies focusing on cancer risk among asbestos-exposed workers in some specific industries in Taiwan were recently published. Yang et al. observed an increased risk of lung cancer mortality in Fengtian, a village in eastern Taiwan. Most of the residents were involved in jade processing industries from 1970 until 1980, and the current exposure level to airborne asbestos in rough grinding was up to 4.7 fibres/mL. The risk of lung cancer was increased with a standardised mortality ratio (SMR) of 1.28 (95% CI 1.12 to 1.45) [12]. In southern Taiwan, workers in shipbreaking industry were thought to be heavily exposed to many possible respiratory carcinogens, including welding fumes, paints, and asbestos [13]. Wu et al. conducted retrospective cohort studies over 4,000 male workers, collected from the Kaohsiung Shipbreaking Worker Union to examine mortality and cancer incidence. Elevated risk of all cancer, oral cancer, and lung cancer was documented among male shipbreaking workers. The flame cutters had highest risk of lung cancer, and one deceased man in this occupation died of pleural cancer [14] younger than 40 years, and two cases of mesothelioma were found by linkage with Taiwan's health insurance database and Taiwan Cancer Registry [15, 16].

The Environmental Protection Administration of Taiwan (Taiwan EPA) promulgated the Toxic Chemical Substances Control Act in 1986, with asbestos being regulated as a hazardous material in 1989 and placed under increasingly stricter controls thereafter. The majority of asbestos used in Taiwan was imported, with the amount rising significantly in the mid-1970s and peaking at 35,000 tons per year in the mid-1980s and then falling significantly in the early 1990s due to new regulations. Less than 5000 tons per year were imported by 2001 [17], and the use of asbestos in construction materials was banned in 2008. On 2 February, 2012, the Taiwan EPA announced the following schedule for a total asbestos ban: from August 1, 2012, the use of asbestos was prohibited for the manufacturing of extruded cement composite hollow panels and construction sealants; from February 1, 2013, the manufacture of asbestos roof tiles was prohibited; and from July 1, 2018, the use of asbestos in the manufacture of brake linings will be prohibited [18].

To the best of the authors' knowledge, the risks of contracting asbestos-related cancers among Taiwanese workers employed in the asbestos factories registered by labour insurance have not yet been documented. This paper thus aimed to determine the SIR of malignant pleural mesothelioma among workers potentially exposed to asbestos from workplace in Taiwan.

## 2. Materials and Methods

### 2.1. Establishment of the Study Cohort

This research was approved by the Institutional Review Board of National Cheng Kung University Hospital (IRB number B-ER-102-162). A total of 389 asbestos-related factories were registered and categorized into seven groups by the Taiwan EPA to produce a roster profile of asbestos-related institutions. The roster profile includes the variables of value added tax (VAT) number, industrial classification, industrial code, industry name, industry address, contact phone number, main products, raw materials, industry registry, and corporation registry. The seven groups are the asbestos-cement industry, asbestos friction industry, asbestos thermal insulation industry, industries using serpentine, those that applied to use asbestos, those that applied to use asbestos but did not actually use it, and not classified.

Workers in the roster profile of asbestos-related institutions were then linked to the labor insurance database. A total of 566,901 pieces of data were obtained, equaling 183,560 people in this cohort.

### 2.2. Airborne Asbestos Concentrations in Asbestos-Related Industries

The asbestos concentrations in the ambient air in and around different factories were obtained from a literature review. We only reviewed studies conducted by governmental agencies or independent research institutes. During the late 1980s to early 2000s, several studies were conducted to measure the airborne asbestos concentrations via personal sampling and area sampling with regards to the maximum risk groups in Taiwan. The related samples were taken from workers in the four main asbestos-related industries of cement, friction, thermal insulation and textiles, and shipyards. The sampling and analytical methods used for asbestos measurement recommended by the Council of Labor Affairs (CLA) are based on those used by the United States National Institute of Occupational Safety and Health (US-NIOSH) and have been validated by several qualified laboratories in Taiwan [19, 20].

### 2.3. Determination of Individual Cancer Diagnosis

Subjects were followed from 1980 through 2009. To assess the incidence of cancer, the personal identification numbers (IDNs) of these workers were encrypted and identified through linkages with the Taiwan Cancer Registry from 1980 to 2009 with all cancers, including MPM coded by the International Classification of Diseases, 9th Revision (ICD-9), as 163 and lung cancer coded as 162.

### 2.4. Statistical Analyses

The National Institute for Occupational Safety and Health (NIOSH) Life Table Analysis System (LTAS) (website link: http://www.cdc.gov/niosh/ltas/) was used to examine cancer incidence. The main analyses used the Taiwanese general population as a reference. In all analyses, the total person-years at risk (PYAR) of 4,021,078 were stratified by gender and calendar year (in five-year increments). Confidence limits for risk measures were estimated based on a Poisson distribution for the observed outcome, with exact limits for outcomes with 10 or fewer occurrences.

The standardised incidence ratio (SIR) was calculated as the ratio of observed malignancies to the expected number of cases estimated using the Taiwan Cancer Registry file (1980–2009) published by the Health Promotion Administration, Ministry of Health and Welfare [21]. For the incident case analyses, PYAR began on the date of cohort inclusion (the earliest being: 1 March, 1950) and ended with the earliest index date or 31 December, 2009. To ensure enough latency of MPM after exposure to asbestos, the PYAR includes only the first employment before 1989 of people in the profile, with follow-up of the incident cases until 2009.

In this cohort, the first employee in the profile was employed from 1 March, 1950. We summed up the total coverage time of labor insurance as the duration of employment and further assumed that it could be used as a proxy of the amount of exposure to asbestos (subdivided into <1 year, 1–10 years, 11–20 years, and >20 years). We also divided the study group into two categories: those with first employment before 1979 as early employment category and those from 1980 to 2009 as the late employment category, to ensure that it was possible to take account of an adequate latency period of 30 years in the early employment category. To address differences in the duration of employment and first period of employment, the male duration-specific and employment-period-specific SIR of mesothelioma were examined using LTAS.

## 3. Results

### 3.1. Descriptive Statistics

There were a total of 160,640 workers in asbestos-related factories available for study, for a total of 4,021,078 PYAR. Three-quarters of these are male, and most were employed in the related factories for less than one year (49.18%), and more than 70% started this working in asbestos-related factories at less than 30 years old (Table 1). The late employment category accounted for 56.18% of the total, with the early employment category accounting for the remaining 43.82%. Most were employed in the asbestos-related factories in the “not classified” category (60.71%), followed by “those that applied to use asbestos but did not actually use it” (12.13%), “those that applied to use asbestos (8.23%), and “asbestos friction industry” (6.65%).

### 3.2. Cause-Specific Incidence Analysis

A total of 6,544 incidences of cancer were observed in the male cohort, and this was not above expectations (SIR = 0.48, 95% CI 0.47 to 0.49) (Table 2). Seventeen incident cases of MPM were observed, which was above expectations in a statistically significant manner (SIR = 2.65, 95% CI 1.54 to 4.25). However, apart from MPM the SIRs of most of the other cancers were significantly below expectations (Table 2). Among the female workers, there were 1,894 incidences of cancer (SIR = 0.80, 95% CI 0.77 to 0.84). An increased risk of cervical cancer was observed, with SIR = 1.73 (95% CI 1.57 to 1.91, *n* = 412). The incidences of Hodgkin's lymphoma (SIR = 1.32, 95% CI 0.27 to 3.87, *n* = 3) and MPM (SIR = 1.67, 95% CI 0.04 to 9.29, *n* = 1) both exceeded expectations, but since these results were only based on a few cases, they did not reach statistical significance. Other cancers with slightly higher incidence than expected (although statistically insignificant) included cancer of the major salivary gland (SIR = 1.04, 95% CI 0.38 to 2.25), nasopharyngeal cancer (SIR = 1.09, 95% CI 0.79 to 1.46), laryngeal cancer (SIR = 1.69, 95% CI 0.35 to 4.95), endometrial cancer (SIR = 1.06, 95% CI 0.84 to 1.32), urothelial cancer (SIR = 1.42, 95% CI 0.88 to 2.17), and thyroid cancer (SIR = 1.32, 95% CI 0.27 to 3.87).

Table 3 shows the risks of MPM by period of first employment and time since first employment. The male workers employed before 1979 had a much higher incidence of MPM (SIR: 3.68, 95% CI: 2.10 to 5.98) compared to those employed after 1979 (SIR: 0.62, 95% CI: 0.00 to 3.48). No incident case of MPM was found before 20 years since first employment. In the early employment category (1950–1979), there was a clear trend that the longer the latency, the more significant the risk: five incident cases of MPM were observed 20 to 30 years since first employment (SIR = 3.65, 95% CI: 1.19 to 8.52), and 11 cases were observed 30 years since first employment (SIR = 4.52, 95% CI: 2.25 to 8.09). As shown in Table 4, the highest incidence of cancer was observed among male workers employed for more than 20 years (SIR = 5.78, 95% CI: 1.19 to 16.89, *n* = 3). Nevertheless, the risk of MPM was also very high among male workers employed for only 1 to 10 years and diagnosed more than 30 years since first employment (SIR = 5.25, 95% CI: 1.70 to 12.24, *n* = 5). For male workers employed less than 1 year, the overall SIR was 2.61 (95% CI: 1.05 to 5.39), with all cases observed having more than 20 years of latency.

The characteristics of seventeen cases of male MPM were shown in Table 5. All of them had histological evidence of MM. The median age at cancer diagnosis was 63 years ranging from 38 to 74. The median latency of MPM was 33.2 years, and all of the patients developed MPM after 20 years. The median age at first employment was 25 years, the youngest one starting his work from 15 years of age. The median duration of employment was 1.7 years with a wide range from 0.1 years to 30 years. The majority (88%) of MPM cases were diagnosed after 2000. Most (16 cases) of them were first employed before 1979, which was classified as the early period in this study. 16 cases were employed in the “not classified” category, and the other was in the industry of insulation.

### 3.3. Airborne Asbestos Concentration in Asbestos-Related Industries

A review of the literature showed that the airborne asbestos concentration varied significantly, depending on the industries examined and when the study was conducted. Current data showed that the maximal airborne asbestos concentration was associated with the asbestos friction industry, at a level of 24.88 fibres/mL after industrial hygiene improvement. Although a few studies found very different results, the airborne asbestos concentration for all industries studied ranged from 2.08 to 6.25 on average, exceeding the permissible exposure level (PEL) of 1 fibre/mL in 2006, now reduced to 0.15 fibre/mL, as announced by the Taiwan EPA (see Supplementary Table 1 in Supplementary Material available online at http://dx.doi.org/10.1155/2015/678598). Both the total number of asbestos-related factories and the number of workers directly exposed in them fell dramatically to about one-fourth to one-third from 1987 to 2000, especially in textile and ground tile factories, in which the use of asbestos was banned by the Taiwan EPA (Supplementary Table 2).

## 4. Discussion

To the best of our knowledge, this is the first retrospective cohort study to explore cancers risk among employees nationwide asbestos-related industries. The cohort started as early as 1950 when the Bureau of Labour Insurance was just established in Taiwan. The highest risk of MPM was observed in workers employed earlier (before 1979) and who had been employed in such factories for more than 20 years. To some extent, these results corroborate an earlier model showing that mesothelioma risk is determined mainly by the time since first employment and minimal duration of employment is sufficient for causing MM given adequate latency [22].

No increase in overall cancer incidence was found in our cohort of workers in asbestos-related factories compared to the general population. In 1974, McMichael coined the term healthy worker effect (HWE) to describe a phenomenon in which more vigorous occupations had a relatively lower mortality rate when compared with the death rate in occupations of an easier character, or among the unemployed [23]. A year later, Goldsmith [24] pointed out that most industrially employed cohorts should be expected to have better life expectancy than unemployed persons. These phenomena are clearly seen in the findings of the current study.

Some industries, which applied for asbestos use but actually did not use it according to the report system of the Taiwan EPA, were included in our nationwide asbestos industries. Thus, a possible dilution of cancer risk in the whole asbestos-related occupational cohort with heterogeneous exposure levels would be likely to cause substantial underestimation of risk of all cancers (Table 2).

Cigarette smoking is a well-established risk factor of developing lung cancer [25]. Previous surveys revealed that the male working population covered by Taiwanese Labour Insurance had a prevalence of cigarette smoking of 48% in 1997 [26], which was lower than 55–60% in the male general population during 1976–1996 [26]. Environmental tobacco smoking (ETS) is also a workplace respiratory carcinogen, and its relative risk of developing lung cancer is estimated to be 3.0 among men [27]. A national workplace smoking survey [27] in 2004 showed that 28.7% of male workers in the manufacturing industries (the majority of asbestos-related factories belonged to this type of industries) reported exposure to ETS, which was lower than that of all male workers in Taiwan (33.4%) [28]. Britain estimate of population attributable fraction (PAF) of occupational asbestos exposure to lung cancer in men was around 9% [29], which implies occupational asbestos may contribute only a minor role compared with smoking. Therefore, using the Taiwanese general population as the reference, significantly decreased risk of lung cancer among workers in the asbestos-related industries might be explained, or at least partially, by healthy worker effect and possibly lower prevalence of smoking habits as well as secondary smoking.

In addition to MPM and lung cancer, asbestos may also be associated with increased risk of laryngeal, pharyngeal, and oral cavity cancers, as well as cancers of GI tract and colorectum [30]. Other malignancies that have been linked to asbestos, albeit inconclusively, include cervical and prostate cancer [31], and some studies have even reported a greater incident risk of brain cancer and testis cancer among male workers employed in asbestos-cement factories [22]. In our study, the SIR of cervical cancer was statistically significantly higher among female workers. This association, however, was not consistent when classified by period of first employment and time since first employment, and thus a causal association for an increased risk of cervical cancer among women exposed to asbestos is less likely. However, more work is required to confirm or refute this statistical association.

Most related studies exclude workers who were exposed to asbestos for less than one year, while our current work included all workers, no matter how long their period of exposure was. This approach is based on prior knowledge and the special circumstances that apply in Taiwan, as follows: (1) the scientific community is in overwhelming agreement that there is no safe level of exposure to asbestos [31]. Moreover, there is no evidence of a threshold level, below which there is no risk of mesothelioma [32]. Minimal exposure to asbestos with long enough latency will thus lead to mesothelioma, and the risk increases exponentially with time since first exposure after the first 10 to 15 years [1], with the greatest risk with regards to mesothelioma occurring approximately 20–40 years later [33]. (2) In Taiwan small- and medium-sized enterprises were once the major part of the economy, and many workers at these were not enrolled in labor insurance at all or were exposed to substantial amounts of asbestos many years before such insurance was offered. Therefore, eliminating the workers with less than one year insurance from the sample was meaningless with regards to guaranteeing adequate exposure in the current study.

According to the Taiwan Cancer Registry file, 54 incident cases of MPM were diagnosed in 2010 [16]. Since more than 90% of the cancers were attributable to asbestos in male workers and about one-quarter in female workers [19], we might expect many cases of MPM in the current study. However, in this 29-year cohort, only 17 male incident cases and 1 female incident case were observed. The huge gap between expectations and actual results may be due to the following reasons: (1) the roster profile of asbestos-related facilities provided by the Taiwan EPA may not include all such places, as many factories continued to use asbestos without being registered, due to management decisions. In addition, the employment in such firms might also be underreported, especially early in the study period. (2) Diagnosis of MM was difficult to identify, due to the lack of a specific code in ICD-9 [34]. For example, in ICD-9 MM can be coded as 158.9 for peritoneal cancer, 162.9 for lung cancer, 163.9 for pleural cancer, and 199.1 for cancer of unspecific site. According to previous studies [20, 35, 36], about 52% to 67% of mesothelioma deaths were coded as 199.1. The site-specific ICD-9 codes for MPM of 163 and 158 (peritoneum) capture only a fraction of all MM deaths in the United States, and in a recent study only 13% of all MM death were coded to ICD-9 163 and 4% coded to 158 [37]. This coding system thus underestimates the incidence of malignant mesothelioma [38–41]. (3) In Taiwan, a population-based cancer registry was established in 1979 that collected basic information, called the short-form system. After 2002, a long-form system was established to collect more detailed information. In 2003, the Cancer Control Act was passed, in which all hospitals were mandated to submit information about cancer patients with trace-back procedure [21], which led to a marked improvement in the quality of cancer registry data [42].

The emergence of asbestos-related diseases has been observed in Taiwanese workers with occupational exposure to asbestos, including (1) pleural plaque in a female worker in asbestos mining in eastern Taiwan [43], (2) pulmonary fibrosis identified among male workers in Taiwanese jade manufacturing factories [12], and 10 patients of asbestosis among male shipbreaking workers [15]. A few cases of MPM in Taiwan have been reported with significant occupational exposure to asbestos, including pipefitter in a shipyard [44] and a 39 year-old carpenter with probable bystander exposure in construction industry [45]. This study with adequate person-years of follow up to detect the risk of MPM corroborated the causal association between occupational asbestos exposure and MPM.

To set up epidemiological surveillance of the effects of asbestos exposure on the French population health, the National Mesothelioma Surveillance Program was established in 1998 in France based on continual following up of MPM [46]. Koreans have begun a nationwide MM surveillance system since 2000 to monitor pathologically confirmed cases and their exposure histories by interview [47]. A clinicopathological correlation study on 1,445 MM cases revealed that the largest number of cases was from shipyard, service in the Navy, the construction industry, and the insulation industry [48]. We also found that patients with MPM were workers employed in the shipyards and insulation industry. Thus, we recommended that MM surveillance system should be established in Taiwan as early as possible for (1) collecting occupational and environmental exposure to asbestos; (2) monitoring trends of epidemiological data; and (3) identifying sentinel cases in high-risk industries for early detection of asbestos-related diseases.

### 4.1. Limitations

There are several limitations in this study that suggest directions for future research, as follows. First, while there were various exposure levels at different factories, the current study lacks a detailed exposure assessment. This is because we did not have data regarding the type of asbestos used in each factory, and measurements of the airborne asbestos dust concentration were based on a literature review. Some studies in the 1980s can provide a rough picture of asbestos exposure in the past. For example, a general survey of 33 registered asbestos factories in Taiwan during 1986-1987 found that all of them employed less than 100 workers, for the average duration of 8.1 years, and thus had a high turnover rate, with a low awareness of the related hazards, and only about 42% of them provided regular medical screening [49]. According to an industrial hygiene survey, chrysotile was in use in the majority of the factories (31/33, 94%), one factory used amosite, and a mixture of chrysotile, amosite, and crocidolite was used in another factory [49]. Nationwide determinations of the airborne asbestos concentration of asbestos-related factories vary in different studies conducted in different periods. Chang et al. [50] used data from 1987 to 1988 and undertook a thorough survey of the ambient asbestos concentrations in the four main asbestos-related industries of cement, friction thermal insulation, and textiles in Taiwan. The concentration of airborne asbestos was the highest, at 6.25 fibres/mL, in asbestos textile factories, followed by the friction factories and insulation factories, with 3.57 fibres/mL and 2.23 fibres/mL, respectively. Some tasks, such as grinding and drilling in the friction industry, had highest concentrations of 10 fibres/mL [49, 51]. While these studies provide valuable information with regards to exposure data, they cover a very broad range of contexts and thus the results are of doubtful generalizability. Second, we did not have data on cigarette smoking among the workers in asbestos-related factories. While smoking is a well-established risk factor associated with lung cancer, it has little or no effect on the incidence rates of mesothelioma [52]. In addition, our results showed no increased risk of lung cancer among the workers. Third, historical rates of asbestos use are associated with a greater risk of mesothelioma approximately 20–40 years later [25]. In this 29-year follow-up study our observation span was limited from 1980 to 2009, which might not be enough due to the long latency of mesothelioma, and more incident cases of MPM are expected after 2009. Further observations are thus essential.

## 5. Conclusions

In this study a higher risk of MPM was found among male workers employed in the early period, before 1979, especially those employed for more than 20 years. Our findings support the view that minimal exposure to asbestos with long latency is enough to increase the risk of MPM.

## Supplementary Material

Based on literature review of seven studies, airborne asbestos concentrations measured during late 1980s to early 2000s were shown in Supplementary Table 1.
Chang et al (2002) reported the number of workers and numbers of different types of factories in 1987 and 2000 in Taiwan, as shown in Supplementary Table 2.

## Figures and Tables

**Table 1:** Demographic characteristics of the cohort, 1950–2009.

	Case number (%)		
	Total	Male	Female
	160640	121883 (75.9)	38757 (24.1)
Asbestos-related industry			
Asbestos cement industry	5919 (3.68)	4965 (4.07)	954 (2.46)
Asbestos friction industry	10681 (6.65)	6745 (5.53)	3936 (10.16)
Asbestos thermal insulation industry	3554 (2.21)	2477 (2.03)	1077 (2.78)
Industries using serpentine	10261 (6.39)	7089 (5.82)	3172 (8.18)
Those applications with use	13220 (8.23)	9630 (7.9)	3590 (9.26)
Those applications without use	19488 (12.13)	11640 (9.55)	7848 (20.25)
Not classified	97517 (60.71)	79337 (65.09)	18180 (46.91)
Period of first employment			
1950~1979	70399 (43.82)	58080 (47.65)	12319 (31.79)
1980~2009	90241 (56.18)	63803 (52.35)	26438 (68.21)
Duration of employment (year)			
<1	78999 (49.18)	59918 (49.16)	19081 (49.23)
1–9	60074 (37.4)	44658 (36.64)	15416 (39.78)
10–19	11572 (7.2)	9137 (7.5)	2435 (6.28)
≥20	9995 (6.22)	8170 (6.7)	1825 (4.71)
First exposure age (years old)			
<30	113202 (70.47)	86025 (70.58)	27177 (70.12)
30–39	28234 (17.58)	20757 (17.03)	7477 (19.29)
≥40	19204 (11.95)	15101 (12.39)	4103 (10.59)

**Table 2:** Observed and expected numbers and standardized incidence ratios for cancers among the male and female workers employed in asbestos-related factories.

Cancer type	Males			Females		
	Obs. N.O.	Exp. N.O.	SIR (95% CI)	Obs. N.O.	Exp. N.O.	SIR (95% CI)
All cancers	6544	13731.1	0.48^*∗∗*^ (0.47–0.49)	1894	2357.8	0.80^*∗∗*^ (0.77–0.84)
Lip, oral cavity, and pharynx	726	1206.1	0.60^*∗∗*^ (0.56–0.65)	20	27.62	0.72 (0.44–1.12)
Major salivary glands	26	29.58	0.88 (0.57–1.29)	6	5.79	1.04 (0.38–2.25)
Nasopharynx	363	439.58	0.83^*∗∗*^ (0.74–0.92)	43	39.62	1.09 (0.79–1.46)
Esophagus	241	378.2	0.64^*∗∗*^ (0.56–0.72)	5	5.62	0.89 (0.29–2.08)
Stomach	409	591.48	0.69^*∗∗*^ (0.63–0.76)	58	76.96	0.75^*∗*^ (0.57–0.97)
Colon, rectum, and anus	872	5186.73	0.17^*∗∗*^ (0.16–0.18)	147	623.28	0.24^*∗∗*^ (0.20–0.28)
Liver and intrahepatic bile ducts	1280	1971.02	0.65^*∗∗*^ (0.61–0.69)	120	143.87	0.83^*∗*^ (0.69–1.00)
Gall bladder and extrahepatic bile ducts	45	87.53	0.51^*∗∗*^ (0.37–0.69)	9	16.64	0.54 (0.25–1.03)
Pancreas	104	167.71	0.62^*∗∗*^ (0.51–0.75)	19	23.64	0.80 (0.48–1.25)
Nose, middle ear, and sinuses	18	34.84	0.52^*∗∗*^ (0.31–0.82)	1	4.14	0.24 (0.01–1.35)
Larynx	86	139.96	0.61^*∗∗*^ (0.49–0.76)	3	1.77	1.69 (0.35–4.95)
Trachea, bronchus, and lung	758	1222.97	0.62^*∗∗*^ (0.58–0.67)	123	144.80	0.85 (0.71–1.01)
Retroperitoneum	6	13.99	0.43^*∗*^ (0.16–0.93)	4	3.95	1.01 (0.28–2.60)
Pleura (malignant mesothelioma)	17	6.41	2.65^*∗∗*^ (1.54–4.25)	1	0.60	1.67 (0.04–9.29)
Bone and articular cartilage	15	19.54	0.77 (0.43–1.27)	2	4.41	0.45 (0.05–1.64)
Skin	205	249.04	0.82^**^ (0.71–0.94)	43	44.04	0.98 (0.71–1.32)
Female breast	—	—	—	494	493.63	1.00 (0.91–1.09)
Uterus and corpus uteri	—	—	—	78	73.51	1.06 (0.84–1.32)
Cervix	—	—	—	412	238.08	1.73^**^ (1.57–1.91)
Adnexa (including ovary)	—	—	—	53	67.28	0.79 (0.59–1.03)
Prostate	307	459.99	0.67^**^ (0.59–0.75)	—	—	—
Bladder	234	310.72	0.75^**^ (0.66–0.86)	19	26.25	0.72 (0.44–1.13)
Kidney	96	118.19	0.81^*^ (0.66–0.99)	11	26.77	0.41^**^ (0.20–0.74)
Renal pelvis and ureter	80	114.29	0.70^**^ (0.56–0.87)	21	14.77	1.42 (0.88–2.17)
Brain	60	86.82	0.69^**^ (0.53–0.89)	15	16.10	0.93 (0.52–1.54)
Thyroid gland	76	91.61	0.83 (0.65–1.04)	91	88.98	1.02 (0.82–1.26)
Hodgkin lymphoma	146	233.45	0.63^**^ (0.53–0.74)	3	2.27	1.32 (0.27–3.87)
Non-Hodgkin lymphoma	6	12.96	0.46 (0.17–1.01)	27	41.66	0.65^*^ (0.43–0.94)
Leukemia	118	165.06	0.71^**^ (0.59–0.86)	22	30.13	0.73 (0.46–1.11)
Others	250	393.35	0.64^**^ (0.56–0.72)	44	71.68	0.61^**^ (0.45–0.82)

^*∗∗*^ *P* < 0.01, ^*∗*^ *P* < 0.05. Obs. N.O.: observed number. Exp. N.O.: expected number; 95% CI: 95% confidence interval.

**Table 3:** Observed and expected numbers and the SIR with the 95% CI for malignant pleural mesothelioma among the male workers employed in asbestos-related factories, by period of first employment in the factory and time since first employment: follow-up for 1980–2009.

Period of first employment	Time since first employment											
	10–20 years			20–30 years			>30 years			Total		
	Obs. (*N*)	Exp. (*N*)	SIR (95% CI)	Obs. (*N*)	Exp. (*N*)	SIR (95% CI)	Obs. (*N*)	Exp. (*N*)	SIR (95% CI)	Obs. (*N*)	Exp. (*N*)	SIR (95% CI)
1950–1979	0	0.45	0.00 (0.00–8.12)	5	1.37	3.65^*∗*^ (1.19–8.52)	11	2.43	4.52^*∗∗*^ (2.25–8.09)	16	4.35	3.68^*∗∗*^ (2.10–5.98)
1980–1989	0	0.54	0.00 (0.00–6.79)	1	0.79	1.26 (0.03–7.03)	0	0.05	0.00 (0.00–70.78)	1	1.6	0.62 (0.00–3.48)

Total period	0	1	0.00 (0.00–3.70)	6	2.16	2.78^*∗*^ (1.02–6.04)	11	2.48	4.43^*∗∗*^ (2.21–7.92)	17	5.95	2.86^*∗∗*^ (1.66–4.57)

^*∗∗*^ *P* < 0.01, ^*∗*^ *P* < 0.05. Obs. (*N*): observed numbers. Exp. (*N*): expected numbers; SIR: standardized incidence ratio; 95% CI: 95% confidence interval.

**Table 4:** Observed and expected numbers and the SIR with the 95% CI for malignant pleural mesothelioma among the male workers employed in asbestos-related factories, by duration of employment in the factory and time since first employment: follow-up for 1980–2009.

Duration of employment	Time since first employment											
	10–20 years			20–30 years			>30 years			Total			
	Obs. (*N*)	Exp. (*N*)	SIR (95% CI)	Obs. (*N*)	Exp. (*N*)	SIR (95% CI)	Obs. (*N*)	Exp. (*N*)	SIR (95% CI)	Obs. (*N*)	Exp. (*N*)	SIR (95% CI)	
<1 year	0	0.49	0.00 (0.00–7.45)	4	1.0446	3.83^*∗*^ (1.04–9.80)	3	0.9784	3.07 (0.63–8.96)	7	2.678	2.61^*∗*^ (1.05–5.39)	
1–10 years	0	0.37	0.00 (0.00–9.85)	1	0.7856	1.27 (0.03–7.09)	5	0.9531	5.25^*∗∗*^ (1.70–12.24)	6	2.2586	2.66 (0.97–5.78)	
11–20 years	0	0.13	0.00 (0.00–28.64)	0	0.1695	0.00 (0.00–21.76)	1	0.196	5.10 (0.13–28.42)	1	0.4943	2.02 (0.05–11.27)	
>20 years	0	0.00	0.00 − 1.5 × 10^8^	1	0.1623	6.16 (0.16–34.34)	2	0.3569	5.60 (0.68–20.24)	3	0.5192	5.78^*∗*^ (1.19–16.89)	

Total	0	1.00	0.00 (0.00–3.70)	6	2.162	2.78^*∗*^ (1.02–6.04)	11	2.4845	4.43^*∗∗*^ (2.21–7.92)	17	5.9501	2.86^*∗∗*^ (1.66–4.57)	

^*∗∗*^ *P* < 0.01, ^*∗*^ *P* < 0.05. Obs. (*N*): observed numbers. Exp. (*N*): expected numbers; SIR: standardized incidence ratio; 95% CI: 95% confidence interval.

**Table 5:** Characteristics of 17 male cases of MPM employed in asbestos-related factories.

Characteristics	Percentage (%)
Histology (ICD-O)^*∗*^	
Mesothelioma, malignant, NOS (9050/3)	64.7
Fibrous mesothelioma, malignant (9051/3)	11.8
Epithelioid mesothelioma, malignant (9052/3)	23.5
Period of cancer diagnosis	
1980–1999	11.8
2000–2009	88.2

Other characteristics	Median (range), years

Age at first employment	25 (15–48)
Age at cancer diagnosis	63 (38–74)
Latency (time since first employment to cancer diagnosis)	33.2 (22.7–56.4)
Duration of employment	1.7 (0.1–30)

^*∗*^Coded by International Classification of Diseases for Oncology- (ICD-O-) 3rd ed., published by WHO Library Cataloguing-in-Publication Data, 2000, reprinted in 2005.
