# Supplementary material for: Increased Standardised Incidence Ratio of Malignant Pleural Mesothelioma in Taiwanese Asbestos Workers: A 29-Year Retrospective Cohort Study
Source: Biomed Res Int. 2015 Jul 28;2015:678598. doi: 10.1155/2015/678598 (PMC4531167; doi:10.1155/2015/678598)
Supplement: Supplementary file 1 — Based on literature review of seven studies, airborne asbestos concentrations measured during late 1980s to early 2000s were shown in Supplementary Table 1. Chang et al (2002) reported the number of workers and numbers of different types of factories in 1987 and 2000 in Taiwan, as shown in Supplementary Table 2. [file 678598.f1.docx]

**Supplementary Table 1** Airborne asbestos concentration in different industries

| Industry type | Asbestos concentration (mean(SD)) (fibers/mL) | | | | | | | |
| --- | --- | --- | --- | --- | --- | --- | --- | --- |
|  | Researcher, published year | | | | | | | |
|  | Lin, 1986[1] | Chang, 1988[2] | Lin, 1994[3] | IOSH84-H305, 1995[4] | IOSH, 1998[5] | Wang, 2002[6] | Tsai, 2003[7] |  |
| Asbestos cement industry |  | 2.13 (2.69)  Range: 0.04-13.84 | 1983-1984: 4.85(3.11), 3.75(5.32), 2.08 (2.26)  1989: 0.36±3.34 |  |  |  | 0.15(0.14) |  |
| Asbestos friction industry |  | 3.57 (1.91)  Range: 1.40-6.18 |  | 2.963 (3.18)  Range: 0.67-11.41 | Range: 0.33-0.79 | 1992: 1.908 (3.397)  Range: 0-24.888  1995: 2.492 (2.789)  Range: 0.027-11.412  1996: 0.11 (0.165)  Range: 0.01-0.3  1998: 0.741 (0.522)  Range: 0.12-1.99 | 0.044 (0.042) |  |
| Asbestos thermal insulation industry |  | 2.23 (0.40)  Range: 2.20-2.25 |  |  |  |  |  |  |
| Asbestos textile industry |  | 6.25 |  |  |  |  |  |  |
| Shipbreaking | 0.18  Range: 0.02-1.0 |  |  |  |  |  |  |  |

**Supplementary Table 2** Numbers of different types of asbestos factories and numbers of workers directly exposed to asbestos in them in 1987 and 2000 in Taiwan [8]

|  | No. of factories | |  | No. of workers | |
| --- | --- | --- | --- | --- | --- |
| Type of factories | 1987 | 2000 |  | 1987 | 2000 |
| Cement | 21 | 6 |  | 298 | 82 |
| Friction | 13 | 7 |  | 119 | 66 |
| Textile | 3 | 0 |  | 28 | 0 |
| Ground tile | 2 | 0 |  | 14 | 0 |
| Insulation | 1 | 1 |  | 8 | 6 |
| Refractory | 1 | 0 |  | 4 | 0 |
| Total | 41 | 12 |  | 471 | 154 |

References:

1. National Science Council (NSC). Investigation of asbestos contamination caused by shipbreaking. NSC-75-0412-B002(25), 1986. Taipei, Taiwan.

2. **Chang HY**, Wang JD, Chang JW, et al. Airborne asbestos concentration in asbestos-related factories in Taiwan. *J Natl Public Health Assoc (R.O.C.)* 1988; **8**: 28-35.

3. **Lin YC**, Xu JH, Ye WY, et al. An Effective Union Organization Pattern for the Management of Asbestos for Small Asbestos Cement Sheet Factories. *J Natl Public Health Assoc (R.O.C.)* 1994;**13**:77-86.

4. Institute of Occupational Safety and Health (IOSH). Exposure to asbestos and its control in barking lining plants: final report. IOSH-H101, 1996. Taipei, Taiwan.

5. Institute of Occupational Safety and Health (IOSH). Safety and health improvement in asbestos work place: final report. IOSH-H101, 1998. Taipei, Taiwan.

6.**Wang GH**, Yu RB, Ye YW, et al. Asbestos Exposure Assessment and Workplace Improvement in the Brake Lining Industry. *Journal of Occupational Safety and Health.* 2002;**10**:98-108

7. **Yeh HJ**, Mao IF, ChenML, et al. Determination of Airborne Asbestos Concentration Nearby the Asbestos-Related Factories by Using Phase Contrast Microscopy. *Chia-Nan Annual Bulletin* 2003;**29**:184-92.

8. **Chang HY**, Wang JD, Chen CW, et al. Asbestos in Taiwan: country report. In: Takahashi K, Lehtinen S, Karjalainen A (eds.). Proceedings of the Asbestos Symposium for the Asian Countries, September 26-27, 2002. Kitakyushu, Japan, J UOEH, 24, 90-4.
